# The role of PYY in improving insulin resistance

**DOI:** 10.3389/fendo.2026.1784709

**Published:** 2026-02-18

**Authors:** Chunyan Liu, Na Ren, Haixin Zhang, Jian Ma

**Affiliations:** 1 Department of Endocrinology, First Affiliated Hospital, Heilongjiang University of Chinese Medicine, Harbin, Heilongjiang, China; 2 School of Graduate Students, Heilongjiang University of Chinese Medicine, Harbin, Heilongjiang, China

**Keywords:** glucose homeostasis, insulin resistance, intestinal hormones, PYY, Y2 receptor

## Abstract

Insulin resistance (IR) is closely related to type 2 diabetes (T2DM) and metabolic syndrome, which poses a serious challenge globally. In terms of IR treatment, peptide YY holds broad therapeutic potential. The study found that the lack of peptide YY (PYY) is closely related to the occurrence of IR. Therefore, PYY plays an important role in glucose homeostasis and improving insulin sensitivity. Specifically, the two primary circulating forms, PYY(1-36) and PYY(3-36), mediate distinct effects through different neuropeptide Y receptors (YRs). PYY(3-36) predominantly acts through the Y2 receptor (Y2R) in the hypothalamus to suppress appetite and in peripheral tissues (adipose, skeletal muscle, liver) to enhance insulin sensitivity. In contrast, PYY(1-36) acts as a broader agonist, with significant effects on pancreatic β-cell protection and insulin secretion fine-tuning via the Y1 receptor. In addition, PYY(3-36) promotes weight loss by suppressing appetite and delaying gastric emptying, thus improving insulin resistance. This review aims to clarify the biological characteristics and signaling mechanism of PYY, and explore its potential applications in improving insulin resistance. Finally, it will explore the new treatment possibilities of PYY for IR and metabolic diseases based on current research progress.

## 1 Introduction

As a core pathological feature of type 2 diabetes (T2DM) and metabolic syndrome, insulin resistance (IR) has become a major global public health challenge. Its essence is that the sensitivity of the target organ to insulin is reduced, so that insulin cannot normally play its physiological function to promote glucose uptake and utilization, thus causing a series of metabolic disorders (1, 2). The pathogenesis of IR is highly complex, involving interactions among genetic susceptibility and environmental factors such as overnutrition and physical inactivity. Its molecular basis includes defects in multiple steps of the insulin signaling transduction pathway, chronic low-grade inflammation, endoplasmic reticulum stress, mitochondrial dysfunction, oxidative stress, dysfunction of adipose tissue, and dysbiosis of the gut microbiota (1).

In recent years, with the deepening study of the gut endocrine system, the gut, as the largest endocrine organ in the human body, has attracted increasing attention for its role in metabolic regulation beyond classical insulin target tissues. Gut hormones not only finely regulate the secretion of pancreatic hormones through the “brain-gut axis,” but also influence central appetite and energy balance regulation via the same pathway, thereby indirectly affecting systemic insulin sensitivity. Among these, PYY, a hormone secreted by intestinal L cells, circulates in two principal bioactive forms: PYY(1-36) and PYY(3-36), which exhibit distinct receptor affinities and physiological roles (3). Initially, PYY(3-36) was highly regarded for its potent appetite-suppressing effects mediated by hypothalamic Y2 receptors, but increasing evidence suggests that both PYY forms participate in glucose homeostasis and insulin sensitivity through complex, often complementary mechanisms, offering new potential therapeutic targets for insulin-resistant diseases (4, 5). This article systematically discusses the biological characteristics of PYY, its interactions with the insulin signaling pathway, and the related mechanisms in improving IR, while integrating preclinical and clinical research evidence to explore the potential and challenges of PYY as a novel therapeutic target.

## 2 Biological properties of PYY

### 2.1 Synthetic secretion and biological expression of PYY

PYY was first isolated and named in 1980 from the small intestine of pigs, with its name derived from its molecular feature of having tyrosine at both the N-terminus and C-terminus (6). During biosynthesis, PYY is a peptide hormone encoded by the PYY gene, which is located on chromosome 17q21.1. After transcription, the precursor protein is processed to generate the active peptide YY (7, 8). PYY mainly circulates in two forms, namely PYY(1-36) and PYY(3-36). Dipeptidyl peptidase IV (DPP-IV) is an enzyme widely expressed on the surface of vascular endothelial cells. It rapidly cleaves the N-terminal Tyr-Pro dipeptide of PYY(1-36), converting it into PYY(3-36) (9, 10). Postprandially, PYY(3-36) becomes the major circulating form, accounting for over 60% of total PYY under fed conditions (11). PYY(1-36) and PYY(3-36) are two forms of PYY with distinct receptor binding properties and biological functions. PYY(1-36) is the full-length form capable of activating multiple Y receptors, especially Y1 receptors, and thus plays a role in protecting pancreatic β cells and inhibiting gastrointestinal motility. PYY(3-36) is the short-chain form generated by cleavage by DPP IV, which is almost exclusively active at Y2 receptors and exerts an appetite-suppressing effect, making it a key molecule in current clinical research for weight control. PYY is cleared mainly via renal excretion and enzymatic degradation, resulting in a short plasma half-life of about 10 minutes in humans and possibly shorter in mice (12, 13). This rapid turnover metabolism requires its analogs to be modified for prolonged action in therapeutic development.

In adult mammals, PYY expression is primarily confined to endocrine cells within the gastrointestinal tract. Its concentration increases along a longitudinal gradient throughout the gastrointestinal tract, peaking in L cells of the colonic and rectal mucosa (3, 14). Notably, intestinal L cells constitute a heterogeneous population that co-expresses multiple hormones. The results suggest that a common PYY-expressing progenitor cell can differentiate into all colonic and intestinal endocrine cells. Approximately 50% of cells expressing glucagon-like peptide-1 (GLP-1), cholecystokinin (CCK), and neurotensin (NT) also co−synthesize PYY. Still, co-expression of PYY with substance P (SP) and 5-hydroxytryptamine (5-HT) in the same cell is very rare in adults (15). This indicates that L cells may develop through different differentiation pathways. In addition to the intestine, PYY is also expressed in small amounts in other tissues. During early embryonic development, pancreatic endocrine progenitor cells generally express PYY, suggesting a potential role in pancreatic development and cell fate determination (16–18). In adulthood, PYY is mainly found in α, δ, and PP cell subpopulations of the pancreas (17, 19, 20). Furthermore, specific regions of the central nervous system (CNS), such as the gigantocellular reticular nucleus in the medulla, contain PYY-positive neurons that project to brainstem areas like the vagal complex and the nucleus of the solitary tract (NTS), participating in the integration of visceral sensation and motor functions (21). In addition to the hypothalamus, brainstem regions, particularly the nucleus of the NTS and the dorsal motor nucleus of the vagus (DMV), serve as key central nodes for PYY signaling. The NTS receives vagal afferent inputs from the gastrointestinal tract, conveying information on nutrient sensing, mechanical distension, and hormone release, which is then integrated and relayed to the hypothalamus and other brain regions to regulate feeding and energy balance (22). PYY-positive neurons located in the medullary gigantocellular reticular nucleus project to the NTS and vagal complex, further contributing to the integration of visceral sensory and motor functions (23). Moreover, vagal afferent terminals express Y2 receptors, and PYY(3−36) can activate these receptors to inhibit vagal activity, thereby delaying gastric emptying and enhancing satiety (24). This brainstem–vagal pathway provides an additional rapid and precise neural circuit underlying PYY−mediated regulation of gastrointestinal motility and feeding behavior.

### 2.2 Synergistic interaction between PYY and GLP-1

PYY and glucagon-like peptide-1 (GLP-1) are both predominantly co-secreted from intestinal L-cells in response to nutrient ingestion, forming a key hormonal duo in the gut-brain metabolic axis. Their interaction extends beyond co-secretion to encompass complementary and synergistic actions at multiple levels. Centrally, PYY(3-36) suppresses appetite primarily via hypothalamic Y2 receptors, while GLP-1 enhances satiety signals through its own receptors in the brainstem and hypothalamus, resulting in a combined anorexigenic effect (25). Peripherally, in the pancreas, PYY(1-36) exerts a fine-tuning, inhibitory effect on insulin secretion via Y1 receptors, which may prevent β-cell overstimulation and exhaustion (26). In contrast, GLP-1 potentiates glucose-dependent insulin secretion (27). This dynamic balance suggests coordinated regulation of insulin output. Furthermore, PYY(3-36) can indirectly enhance insulin sensitivity by promoting GLP-1 secretion, which in turn activates hepatic and peripheral insulin signaling pathways (28). This multi-level functional synergy underscores that PYY and GLP-1 act as interdependent partners in the integrated control of energy homeostasis and glucose metabolism, providing a strong rationale for exploring multi-target therapeutic strategies.

### 2.3 Triggering and regulation of PYY secretion

The secretion of PYY (3-36) has a precise and complex mechanism for regulation. Postprandial plasma PYY (3-36) levels rise rapidly, and detectable changes usually occur within 15–30 minutes, peak after 1–2 hours, and remain at elevated levels for up to 6 hours, reflecting its continuous metabolic regulation after meals (29). Dietary intake is the strongest physiological factor that stimulates PYY (1-36) secretion. Different constant nutrients have different effects on stimulating PYY secretion. Dietary fat is the most effective promoter of PYY secretion, followed by protein. At the same time, carbohydrates have a relatively weaker stimulatory effect (30). The caloric density of food intake is positively correlated with the amplitude of PYY (3-36) release (31). PYY is rapidly released before the chyme reaches the distal intestine, suggesting the presence of neural or hormonal-mediated anticipatory reflexes involving the vagus nerve pathway, cholinergic mechanisms, and upper gastrointestinal hormones such as CCK (32, 33). Meanwhile, gastric distension can also influence PYY release (34). Other regulatory factors, such as bile acids, can stimulate the release of GLP-1 and PYY by activating the TGR5 receptor on the surface of intestinal L cells (35–37). Additionally, short-chain fatty acids (SCFAs) produced by gut microbiota metabolism are believed to promote PYY expression and secretion (38, 39). In pathological conditions, the secretion pattern of PYY changes. For example, in obese individuals, the level of PYY on an empty stomach or after meals is usually reduced or weakened. On the contrary, PYY (3-36) levels may increase in diseases associated with significant weight loss, such as anorexia nervosa and inflammatory bowel disease (40, 41).

### 2.4 Signaling pathways and signal transduction of PYY

PYY plays its biological role by binding to its specific receptor. The Y receptor belongs to the G protein-coupled receptor (GPCR) family. At present, a variety of subtypes have been identified, including Y1, Y2, Y4, and Y5 receptors. The Y6 receptor is a false gene of human beings (42, 43). PYY (1-36) and PYY (3-36) showed significant differences in affinity for Y receptor subtypes (44). PYY (1-36) is a relatively broad-spectrum agonist that can bind to Y1, Y2, Y4, and Y5 receptors, but has different affinity for each subtype. Due to the N-terminal truncation, PYY (3-36) shows high selectivity and affinity for Y2 receptors (45), retaining part of the activity on Y5 receptors (46), but the binding ability to Y1 and Y4 receptors is greatly reduced (47).

**Figure 1:**
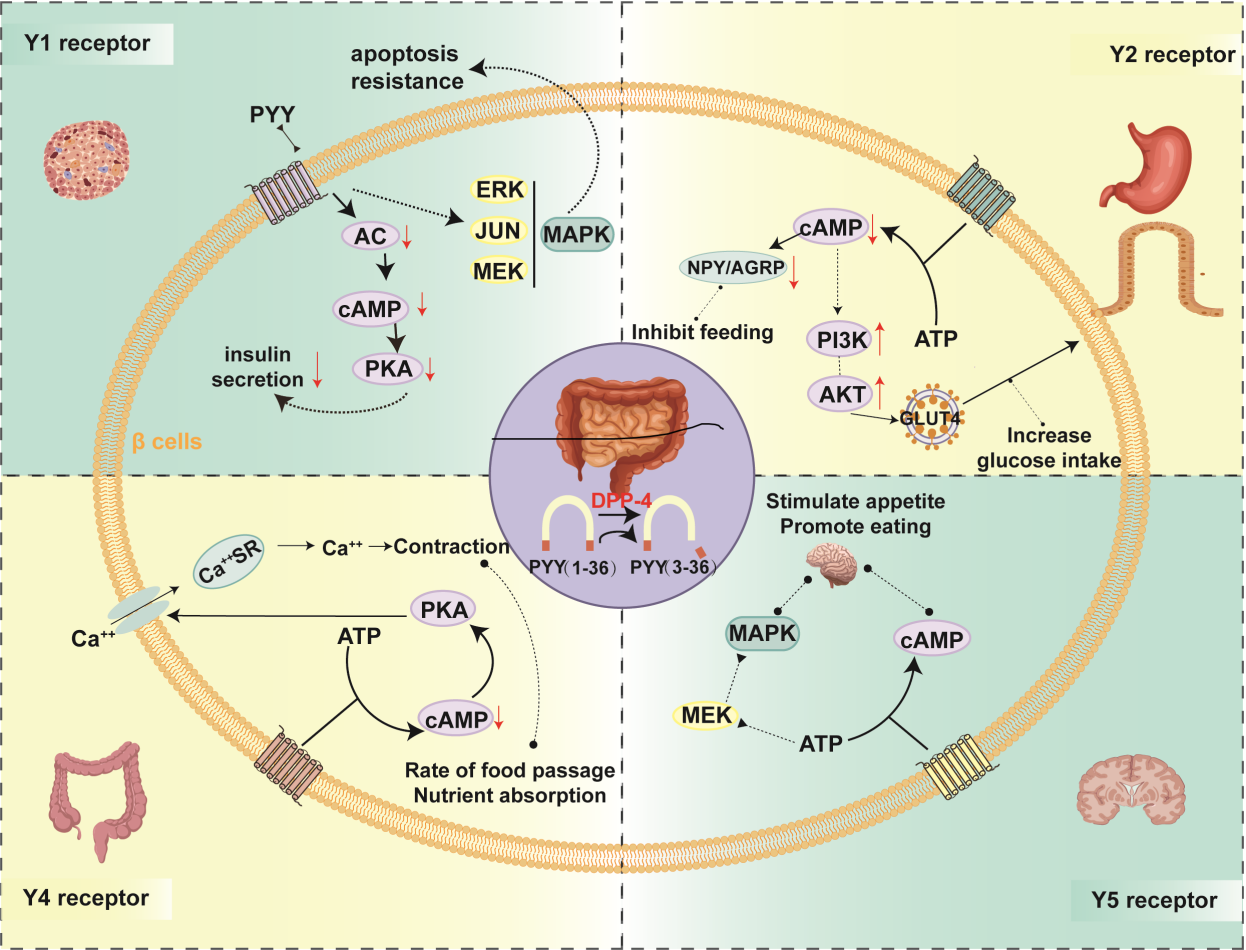
Biological effects of PYY binding to different receptors. This schematic summarizes the distinct signaling pathways and physiological outcomes triggered by the two primary circulating forms of PYY(1-36) and PYY(3-36) upon engaging different neuropeptide Y receptor subtypes (Y1, Y2, Y4, Y5). Activation of the Y1 receptor, primarily by PYY(1-36), in pancreatic β-cells inhibits glucose-stimulated insulin secretion via reduced intracellular cAMP and promotes cell survival and proliferation through the MAPK/ERK pathway. The Y2 receptor, selectively activated by PYY(3-36), mediates appetite suppression in the hypothalamus by inhibiting NPY/AGRP neurons and enhances peripheral glucose uptake via GLUT4 translocation. Binding of PYY(1-36) to Y4 receptors in the gut modulates smooth muscle contraction and delays gastric emptying, thereby slowing nutrient absorption. Meanwhile, hypothalamic Y5 receptor activation promotes feeding behavior through Gi/o-mediated cAMP inhibition. Collectively, these receptor-specific mechanisms underlie PYY’s integrated regulation of energy balance, glucose homeostasis, and insulin sensitivity across multiple tissues. AC, Adenylate cyclase; cAMP, cyclic adenosine monophosphate; PKA, protein kinase A; ATP, adenosine triphosphate; ERK, extracellular signal-regulated kinase; JUN, Jun proto-oncogene, AP-1 transcription factor subunit; MEK, mitogen-activated ERK-activating kinase (MAP kinase kinase); MAPK, mitogen-activated protein kinase; NPY, neuropeptide Y; AGRP, agouti-related neuropeptide; PI3K, phosphoinositide 3 kinase.

The distribution of Y receptors in different tissues determines the diversified physiological functions of PYY (**Figure 1**). Y1 receptors are widely expressed and are mainly concentrated in pancreatic islets (especiallyβ, δ, andα cells), vascular smooth muscle, and specific nuclei in the cerebral cortex, amygdala, and hypothalamus. It mainly mediates the inhibitory effect of PYY (1-36) on insulin secretion and the vasoconstrictive effect (48–50). They inhibit adenylate cyclase (AC) onβ cells and reduce adenosine cyclophosphate (cAMP), thus reducing the activity of cAMP-dependent protein kinase (PKA). The cAMP-PKA pathway is a key component that promotes the evocitation of insulin particles; inhibiting this pathway can reduce insulin oversecretion and prevent compensatory insulin resistance caused by persistently high insulin levels in β-cells (20). In addition, PYY(1-36) can regulate beta cell proliferation and prevent cell apoptosis by activating the filamentogen-activated protein kinase (MAPK) signaling pathway, thus retaining the quality of β-cells (51, 52). Research evidence shows that Y1 receptor agonists can prevent obesity in female mice and enhance β-cells function in obesity-induced diabetes models (53). Moreover, activation of Y1 receptors in α cells can promote the transdifferentiation of α cells into β cells, increasing β cell mass (53, 54).

Y2 receptors are primarily expressed in the CNS, including the arcuate nucleus of the hypothalamus, the solitary tract nucleus, and posterior regions of the brainstem, enteric neurons, and peripheral nerve endings (55, 56). Y2 receptors are high-affinity autoreceptors for neuropeptide Y (NPY) and PYY (3-36), belonging to the Gi/o-coupled receptor family. As a major presynaptic autoreceptor, it inhibits cyclic adenosine monophosphate (cAMP) levels by activating Gi/o proteins, thereby reducing NPY/AGRP release and suppressing feeding behavior. This receptor mediates the anorectic effect of PYY (3-36) and inhibits gastric emptying (57, 58). In peripheral adipose tissue, Y2 receptor activation inhibits lipolysis and reduces free fatty acid (FFA) release. In skeletal muscle, Y2 receptor activation inhibits adenylyl cyclase (AC), lowering intracellular cAMP levels. This cAMP reduces the indirect activation of the PI3K-Akt signaling pathway and promotes the movement of vesicles containing GLUT4 from the storage pool to the cell membrane (59), thus increasing glucose uptake. Research shows that the sensitivity of Y2 receptor knockout (Y2R-) mice to anorexia and insulin sensitization of PYY (3-36) is reduced, confirming the core role of the Y2 receptor (60, 61). Presynaptically, Y2 receptors function as autoinhibitory receptors regulating neurotransmitter release, participating in negative feedback control of energy balance.

Y4 receptors are widely distributed in peripheral tissues, particularly showing significant expression in the human small intestine, ileum, coronary arteries, and cardiac tissue (62). Y4 receptors have the highest affinity for PYY from the insulin family and are Gi/o-coupled receptors. Their activation by ligands such as PYY(1-36) inhibits cAMP and elevates Ca²^+^, jointly regulating intestinal smooth muscle tone and secretion, affecting gastric emptying and colonic motility (63). In the gastrointestinal neuroendocrine system, Y4 receptors together with Y2 receptors modulate smooth muscle contraction patterns, influencing food transit rate and nutrient absorption.

Y5 receptors are primarily distributed in the CNS, with particularly high levels detected in the arcuate nucleus and paraventricular nucleus (PVN) of the hypothalamus (64). They were once thought to be closely associated with feeding regulation. Still, studies have confirmed that they promote feeding by inhibiting cAMP via Gi/o receptors and affecting the appetite control network through MAPK ERK in the hypothalamus (51, 64). Y5 receptor defects lead to increased food intake in mice, accompanied by compensatory changes in the expression of AgRP, CART, and POMC genes in the hypothalamus, further confirming their role in regulating energy intake (65). PYY(1-36) and PYY (3-36) mediate signal conduction across multiple tissues and pathways through four GPCR subtypes (Y1, Y2, Y4, and Y5). The core mechanism involves Gi/o-mediated cAMP inhibition, accompanied by branch signals such as MAPK/ERK, PKC-Ca2^+^, and beta-inhibitor-mediated receptorization, which leads to complex tissue-specific cell responses. These reactions eventually improve insulin resistance by regulating various physiological functions, including insulin secretion, vascular tension, gastric emptying, intestinal peristalsis, and eating behavior.

## 3 Insulin resistance

Insulin resistance (IR) is characterized by a reduced physiological response of insulin-sensitive tissues (including liver, skeletal muscle, and adipose tissue) to circulating insulin, although the insulin concentration is normal or even increased (66). This situation will impair insulin-mediated glucose uptake and metabolic regulation. Under normal physiological conditions, insulin maintains glucose homeostasis by promoting the uptake of glucose by cells, promoting the synthesis of glycogen in the liver and muscles, and inhibiting glycogenesis in the liver. In the case of insulin resistance, insulin cannot effectively exert these functions. To maintain blood glucose homeostasis, the body must secrete excessive insulin, resulting in “hyperinsulinemia” (1, 67). Physiologically, IR is defined as a state of reduced responsiveness to high physiological levels of insulin in insulin-targeted tissues. Specifically, at normal plasma insulin levels, target tissues fail to initiate a coordinated hypoglycemic response, including suppression of endogenous glucose production, inhibition of lipolysis, promotion of cellular uptake of plasma glucose, and enhancement of net glycogenesis (68). Persistence of this pathological state over time further burdens pancreatic β-cells, potentially leading to β-cell functional failure and the onset of T2DM (69).

### 3.1 Insulin signaling pathway and key nodes in IR formation

**Figure 2:**
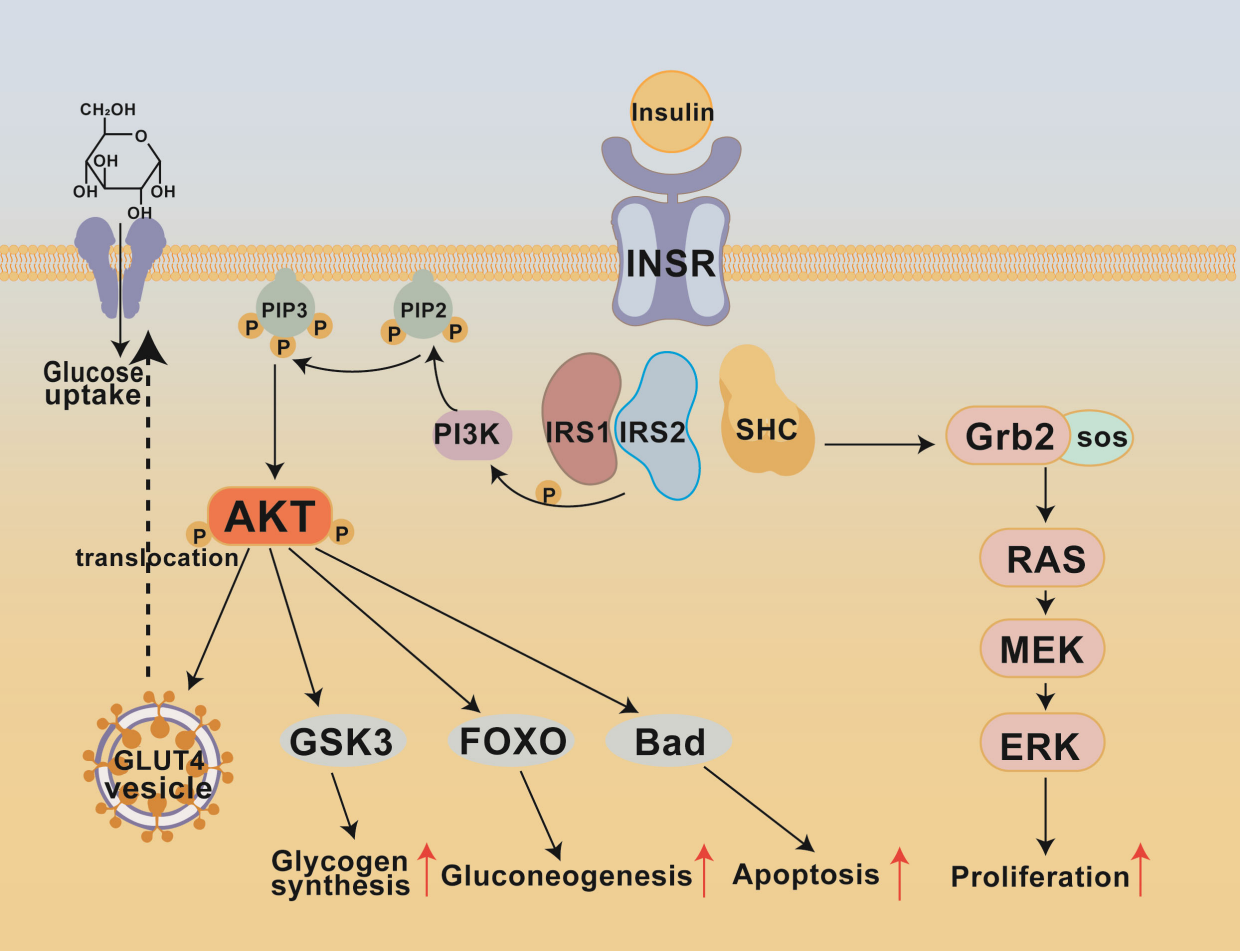
Key signaling pathways in insulin resistance formation. This schematic illustrates the key signaling pathways involved in insulin resistance (IR) formation, depicting the molecular cascade initiated by insulin binding to its receptor (INSR) and the subsequent divergent metabolic and proliferative responses mediated primarily through the PI3K-AKT and MAPK (RAS-MEK-ERK) axes, which coordinately regulate critical cellular processes such as glucose uptake, glycogen synthesis, gluconeogenesis, and cell survival. IRS, Insulin Receptor Substrate; PI3K, Phosphoinositide 3−kinase; PIP2, Phosphatidylinositol-4,5-bisphosphate; PIP3, Phosphatidylinositol-3,4,5-trisphosphate; AKT, serine/threonine kinase; GSK3, Glycogen Synthase Kinase-3; FOXO, Forkhead box O transcription factor; BAD, Bcl-2-associated death promoter; GRB2, Growth factor receptor-bound protein 2; SOS, Son of Sevenless; RAS, Rat sarcoma virus GTPase; MEK, MAP kinase/ERK kinase; ERK, Extracellular signal-regulated kinase.

Insulin initiates its metabolic effects by binding to the insulin receptor (INSR) on target cell membranes (**Figure 2**). INSR is a transmembrane receptor tyrosine kinase organized as an α_2_β_2_ tetramer, with α subunits located extracellularly for ligand binding and β subunits spanning the membrane and harboring the tyrosine kinase domain (70, 71). Insulin binding induces a conformational change in the receptor, exposing the activation loop of the kinase domain. This subsequently triggers autophosphorylation at key tyrosine sites such as Tyr1158, Tyr1162, and Tyr1163, forming high-affinity SH2-binding sites (72). The IRS family includes IRS1 and IRS2, which are key proteins in insulin receptor signaling and intracellular signal transduction (73). Phosphorylated IRS proteins then recruit the p85 regulatory subunit of PI3K via their phosphorylated tyrosine residues, positioning the p110 catalytic subunit near phosphatidylinositol 4,5-bisphosphate (PIP_2_) on the cell membrane to catalyze its phosphorylation at the D3 site, generating the second messenger phosphatidylinositol 3,4,5-trisphosphate (PIP_3_) (74–76). Accumulation of PIP_3_ attracts downstream effector proteins containing PH domains, including PDK1 and mTORC2 (also called PDK2), which phosphorylate Akt (PKB) at Ser308 and Thr473, respectively, leading to full activation of Akt (74, 76). Activated Akt subsequently phosphorylates a series of downstream targets, such as AS160/TBC1D4, promoting the translocation of GLUT4 protein to the plasma membrane and significantly enhancing glucose uptake (74); it also inhibits glycogen synthase kinase 3 (GSK3), relieving the inhibition of glycogen synthase and thus promoting glycogen synthesis in the liver and muscle (74, 76); Akt can also phosphorylate the transcription factor forkhead box protein O1 (FOXO1), inhibiting the transcription of gluconeogenic genes and thereby reducing hepatic glucose production (74, 76). In addition to the PI3K-Akt axis, the insulin IRS complex can activate the MAPK signaling pathway through the Shc-Grb2-SOS-Ras-Raf-MEK-ERK cascade, participating in proliferative effects such as cell proliferation and protein synthesis (77). It coordinates the uptake, storage, and metabolism of glucose, while inhibiting glycogenesis and lipolysis.

When any component of the insulin signaling pathway fails, it can lead to insulin resistance. When there are defects in INSR, including decreased expression of INSR on the surface of cells and decreased activity of receptor tyrosine kinase, decreased INSR levels in obese mice and human fat cells, and decreased IRK activity in diabetic rats (78–80). Ubiquitin ligase (MARCH 1) can reduce the number of receptors on the surface of cells by ubiquitinating INSR, and its expression in the white adipose tissue (WAT) of obese individuals is increased, further exacerbating receptor defects (81). IRS protein also has dysfunction, and the level of IRS1/2 tyrosine phosphorylation is reduced, resulting in impaired PI3K activation. Chronic inflammation, oxidative stress, and other factors can also induce IRS protein serine/threonine phosphorylation and inhibit its normal signaling function (82–84). Impaired activation of downstream signal molecules will lead to a decrease in the level of Akt phosphorylation, thus preventing its full activation and resulting in ineffective GLUT4 transfer regulation, limiting glucose uptake, reducing GYS3 activity, and hindering glycogen synthesis. Moreover, FOXO1 remains unphosphorylated, leading to upregulation of key genes involved in gluconeogenesis and promoting hepatic glucose production (85, 86). The dysregulation of these pathways accumulates, ultimately causing decreased glucose uptake, impaired glycogen synthesis, and enhanced gluconeogenesis, forming the core pathological basis of metabolic disorders such as T2DM.

## 4 Mechanism of PYY improving insulin resistance

**Figure 3:**
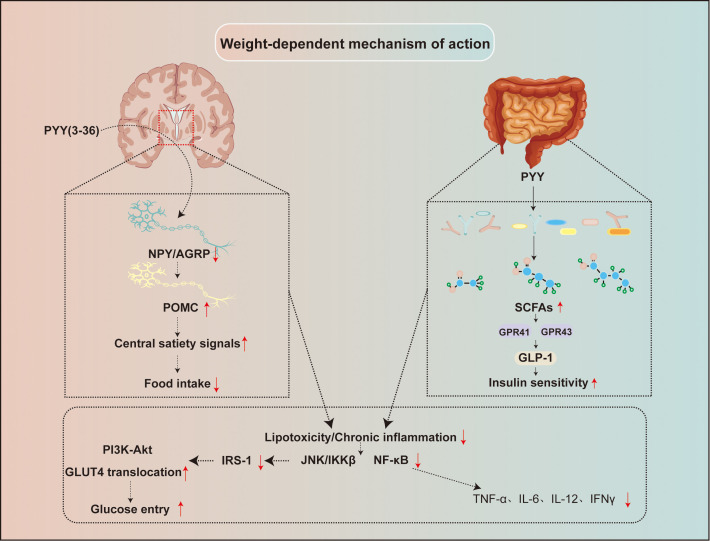
Central and peripheral mediated indirect mechanisms of PYY. This figure illustrates the weight-dependent (indirect) mechanisms by which PYY improves insulin resistance. PYY(3-36) suppresses appetite centrally by inhibiting NPY/AGRP neurons and stimulating POMC neurons in the hypothalamus, thereby reducing food intake. Simultaneously, PYY promotes the production of short-chain fatty acids (SCFAs) by gut microbiota; SCFAs act via GPR41/43 receptors to stimulate GLP-1 secretion, which in turn enhances PI3K-Akt signaling, GLUT4 translocation, and systemic insulin sensitivity. The resulting weight loss lowers circulating free fatty acids and attenuates the release of pro-inflammatory cytokines (e.g., TNF-α, IL-6), thereby relieving lipotoxicity and chronic inflammation-mediated inhibition of insulin signaling pathways such as IRS-1 serine phosphorylation. NPY, Neuropeptide Y; AGRP, Agouti-related protein; POMC, Proopiomelanocortin; GLUT4, Glucose transporter type 4; SCFAs-Short-chain fatty acids.

**Figure 4:**
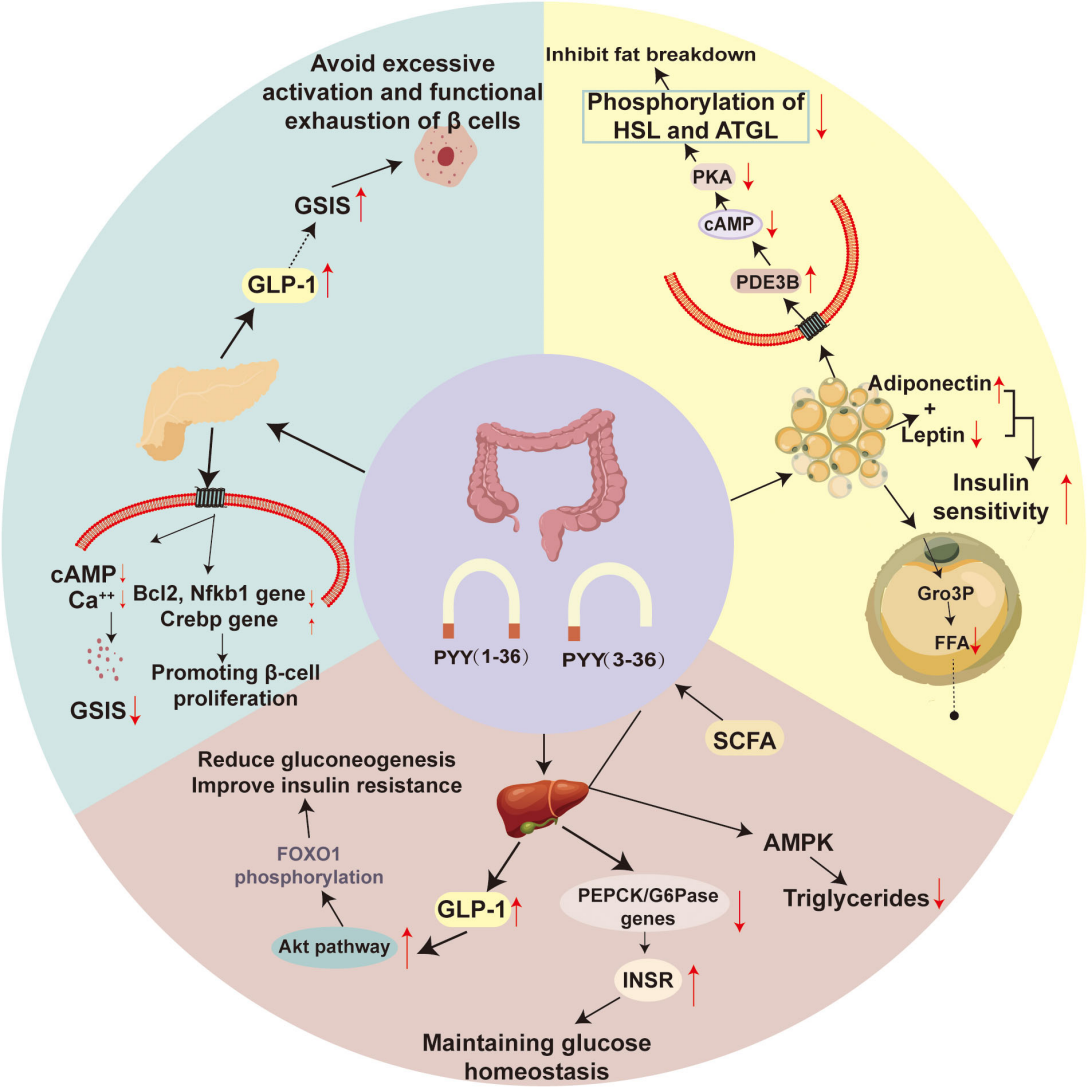
Direct mechanisms of PYY action in the pancreas, liver, and adipose tissue. Illustrates the direct, non-weight-dependent mechanisms through which PYY improves insulin resistance by acting on key metabolic organs. In the pancreas, PYY(1-36) protects β-cell function by fine-tuning insulin secretion and preventing excessive activation, while PYY(3-36) promotes GLP-1 secretion to support insulin release. In the liver, PYY enhances insulin receptor (INSR) expression and activates the Akt pathway, leading to FOXO1 phosphorylation and suppressed gluconeogenesis, thereby maintaining glucose homeostasis. In adipose tissue, PYY(3-36) inhibits lipolysis by reducing cAMP levels via PDE3B activation, which decreases the phosphorylation of HSL and ATGL, limits free fatty acid (FFA) release, and promotes glycerol-3-phosphate (Gro3P) production for triglyceride synthesis. Additionally, PYY modulates adipokine secretion, favoring an improved adiponectin/leptin ratio, which further enhances systemic insulin sensitivity. GSIS, Glucose-Stimulated Insulin Secretion; GLP-1, Glucagon-like peptide-1; INSR, Insulin Receptor; PDE3B, Phosphodiesterase 3B; Gro3p, Glycerol-3-phosphate; FFA, Free Fatty Acids.

PYY improves insulin resistance not through a single mechanism, but via a complex network of regulatory pathways involving the central nervous system and multiple peripheral metabolic organs. These mechanisms can be broadly categorized into weight-dependent (indirect) mechanisms (**Figure 3**) and non-weight-dependent (direct) mechanisms (**Figure 4**).

### 4.1 Weight-dependent (indirect) mechanism of action

Central-mediated feeding suppression and weight loss remain the most classic indirect mechanism of PYY (3-36). Peripherally administered PYY(3-36) crosses the blood-brain barrier to act on Y2R-expressing NPY/AgRP neurons in the hypothalamic arcuate nucleus, inhibiting their activity and enhancing satiety signals (60, 87, 88). Beyond the hypothalamic arcuate nucleus, the brainstem nucleus of NTS plays a significant role in mediating the metabolic effects of PYY(3−36). As a primary integration center for visceral sensory information, the NTS receives vagal inputs from the gastrointestinal tract and expresses abundant Y2 receptors. Activation of Y2 receptors in the NTS by PYY(3−36) can inhibit local GABAergic neuronal activity, enhance glutamatergic transmission, and thereby modulate autonomic outputs to downstream regions such as the rostral ventrolateral medulla (RVLM) and the hypothalamus (89). Neural circuits between the NTS and the dorsal motor nucleus of DMV are involved in the fine-tuning of gastric emptying and intestinal motility (90). PYY acts through this pathway to retard gastrointestinal transit, further promoting satiety and body-weight control. These brainstem mechanisms operate in concert with hypothalamic pathways, together forming a multi−level neural network that underpins PYY’s regulation of energy balance. It may further reduce appetite by decreasing plasma ghrelin levels (91, 92). Chronic administration reduces food intake, leading to an energy deficit, weight loss, and reduced adipose mass. This decrease in adiposity lowers circulating levels of free fatty acids (FFA) and inflammatory mediators, which are key drivers of impaired insulin signaling (93).

Lipotoxicity and chronic inflammation are central to the pathogenesis of insulin resistance. Excess FFA and TNF-α, IL-6 activate pathways involving TLR4, JNK, IKKβ, and NF-κB. These converge to induce serine phosphorylation of IRS-1, which inhibits its tyrosine phosphorylation and subsequent activation of the PI3K-Akt pathway, ultimately hindering GLUT4 translocation and glucose uptake (94, 95). Weight loss, achieved via PYY (3-36)-mediated appetite suppression, reduces FFA flux and inflammatory tone, thereby alleviating this block in insulin signaling and improving systemic insulin sensitivity.

PYY, particularly the truncated form PYY(3-36), is a key regulator of gastrointestinal motility, significantly delaying gastric emptying and small intestinal transit (96, 97). This leads to a more gradual nutrient absorption, blunting postprandial glucose excursions and reducing excessive β-cell stimulation. Slower transit may also prolong nutrient contact with distal L-cells, potentially amplifying the secretion of beneficial hormones like GLP-1, which enhances insulin secretion and further suppresses appetite (98, 99). Furthermore, PYY (3-36) contributes to weight regulation by influencing gut microbiota composition and intestinal barrier function. It promotes *Bifidobacterium* that produces short-chain fatty acids (SCFAs) (38). SCFAs can enhance insulin sensitivity and stimulate GLP-1 secretion (100) and regulate metabolic gene expression by inhibiting histone deacetylase (HDAC) (101). This interplay highlights a positive feedback loop where PYY-enhanced SCFA production further augments the secretion of its co-hormone GLP-1, thereby amplifying the overall satiety and metabolic benefits. PYY (3-36) also supports intestinal epithelial integrity by upregulating tight junction proteins, reducing endotoxin translocation, and alleviating metabolic endotoxemia and chronic inflammation (102, 103). PYY deficiency disrupts microbial balance, while its restoration improves metabolic parameters (104–106). PYY’s effects are often synergistic with other gut hormones. Co-secreted with GLP-1, their combined administration produces stronger anorectic and metabolic benefits than either alone (107). PYY also counterbalances the orexigenic hormone ghrelin (108), and restoring PYY levels can help re-establish energy balance (109).

Therefore, PYY (3-36) not only reduces body weight by decreasing food intake through the CNS but also contributes to weight loss by regulating the gut microbiota and increasing the secretion of beneficial intestinal hormones. When body weight decreases, the levels of FFA and inflammatory factors in the body are reduced, alleviating the impact of lipotoxicity and chronic inflammation on insulin signaling, restoring insulin sensitivity, and improving IR status. Thus, PYY (3-36) plays an important role in both enhancing insulin sensitivity and protecting β-cell function.

### 4.2 Non-weight-dependent (direct) mechanism

PYY acts on the pancreas to protect β-cell function and regulate insulin secretion. Pancreatic β-cell dysfunction is a critical step in the progression of insulin resistance to type 2 diabetes mellitus. In rodents, extensive *in vitro* and *in vivo* studies indicate that PYY (1-36) directly inhibits glucose-stimulated insulin secretion by activating Y1 receptors on β-cells and δ-cells, while PYY (3-36) indirectly enhances insulin secretion by promoting GLP-1 secretion, thereby preventing β-cell overactivation and functional exhaustion. This was once considered PYY’s primary role in the pancreas (110). This inhibitory effect of PYY(1-36) may be achieved by reducing intracellular cAMP levels, influencing calcium signaling, or affecting membrane potential (111). Physiologically, this can be interpreted as a “fine-tuning” or “braking” mechanism preventing excessive postprandial insulin secretion, thereby helping maintain β-cell functional reserve and avoiding hypoglycemia risk (111, 112). Concurrently, PYY (1-36) activates the Y1 receptor to regulate the expression of apoptosis-related genes (e.g., Bcl2, Nfkb1) and proliferation-related genes (e.g., Crebp), thereby promoting β-cell proliferation and protecting β-cells from damage caused by stressors such as STZ, high-sugar, and high-fat diets (113, 114). Research shows that PYY (1-36) and PYY (3-36) can promote the proliferation of permanentβ cell lines and primary pancreatic islets, and protect them from damage from apoptosis inducers such as streptomycin (STZ) (115, 116). In addition, NF-κB regulates PYY gene expression, indicating that NF-κB participates in the PYY-mediated cell survival pathway (117). In the diabetic rat model, continuous exposure to PYY can restore pancreatic insulin secretion (118). This protective effect is essential for maintaining beta cell quality under IR and diabetes conditions. In addition, the extensive expression of PYY during the development of the embryonic pancreas indicates that it may be involved in the determination of cell fate (19). Therefore, PYY (1-36) may protect the quality of β-cells by activating Y1 receptors to reduce insulin secretion, thus preventing apoptosis caused by excessive insulin secretion of pancreatic β-cells and promoting the proliferation of permanent β-cells. Further research shows that the activation of pancreatic Y1 receptors may promote the transdifferentiation of alpha cells into β-cells and increase the number of β-cells, which provides new insights into maintaining the number of β-cells (54, 119).

PYY acts on the liver to inhibit gluconeogenesis and improve lipid metabolism (120, 121). PYY (3-36) activates hypothalamic Y2 receptors, suppressing sympathetic outflow (norepinephrine), which downregulates hepatic gluconeogenic enzymes (G6PC, PEPCK) (122–124). It may also promote GLP-1 secretion, which activates hepatic Akt, leading to FOXO1 phosphorylation and further suppression of gluconeogenesis (39, 125, 126). PYY upregulation reduces the expression of SREBP1c, PPARγ, and e ACC1, thereby inhibiting hepatic triglyceride synthesis (127). It also increases hepatic insulin receptor (INSR) surface expression, enhancing insulin binding and utilization (4, 128). Thus, PYY inhibits hepatic glycogenolysis via the CNS, reduces hepatic fat accumulation, and increases INSR expression to treat insulin resistance.

PYY acts on adipose tissue primarily via Y2 receptors to inhibit lipolysis (129, 130). Activation of Y2R increases phosphodiesterase 3B (PDE3B) activity, lowering cAMP levels and inhibiting PKA, which reduces the phosphorylation and activity of hormone-sensitive lipase (HSL) and adipose triglyceride lipase (ATGL) (131, 132). This decreases FFA release into circulation. Hyperinsulinemic-euglycemic clamp studies show PYY(3-36) reduces plasma FFA turnover in insulin-resistant individuals (133). PYY (3-36) also promotes glucose uptake in adipocytes, providing glycerol-3-phosphate for fatty acid re-esterification, further limiting FFA efflux (133, 134). Additionally, PYY influences adipokine secretion. It is positively correlated with adiponectin levels, an insulin-sensitizing hormone that activates AMPK signaling (135, 136). Conversely, PYY can reduce leptin secretion (137). Conversely, PYY can reduce leptin secretion (130). Lower leptin levels alleviate its inhibitory effects on insulin secretion and improve hepatic insulin sensitivity, while also reducing leptin-driven inflammation (135–137). This shift in the adiponectin/leptin ratio contributes to enhanced systemic insulin sensitivity.

PYY can regulate the expression of pancreatic beta cell apoptosis-related genes, promote beta cell proliferation, and protect beta cell function and reserve by inhibiting insulin oversecretion, thus preventing beta cell dysfunction. At the same time, PYY (3-36) inhibits liver glycogenesis, reduces fat accumulation, increases the expression of INSR in the liver, enhances insulin utilization, and improves insulin resistance. In adipose tissue, PYY (3-36) inhibits lipolysis, promotes lipid storage, and enhances systemic insulin sensitivity by regulating the secretion of fat factors such as adiponectin and leptin, thus improving insulin resistance and glucose homeostasis.

In summary, PYY improves its own metabolism and insulin resistance through a variety of mechanisms. On the one hand, it plays a role through weight-dependent pathways, reducing food intake in the central nervous system and reducing the level of growth hormone-releasing peptides, while delaying gastric emptying and synergizing with GLP-1 to promote weight loss. On the other hand, PYY affects multiple target organs through a direct mechanism unrelated to weight. It protects the function of pancreatic β-cells by promoting β-cell proliferation and inhibiting insulin oversecretion, thus preventing β-cell depletion. It also directly inhibits liver glycolysis, reduces liver fat accumulation, enhances liver INSR expression, and improves insulin utilization. PYY also inhibits lipolysis and improves insulin sensitivity by regulating the secretion of adiponectin and leptin. These multiple effects of PYY effectively reduce the levels of FFA and inflammatory factors, reduce the impact of lipotoxicity and chronic inflammation on insulin signaling pathways, restore insulin sensitivity, maintain glucose homeostasis, and improve insulin resistance. Therefore, PYY plays an indispensable role in improving obesity, metabolic syndrome, and insulin resistance, and its development as a therapeutic agent for insulin resistance shows great promise.

## 5 Integration of experimental and clinical research evidence

### 5.1 Preclinical research evidence

PYY gene knockout (PYY^-^/^-^) mice provide the most direct evidence of PYY’s physiological functions. Multiple independent studies have reported that PYY-/- mice exhibit hyperinsulinemia and are prone to developing obesity and insulin resistance during aging or under long-term high-fat diet challenges (138, 139). These phenotypes strongly suggest that a lack of endogenous PYY is a causative factor in metabolic disorders. Notably, PYY-/- mice show increased POMC mRNA expression in the hypothalamic arcuate nucleus (138), leading to the production of more α-melanocyte-stimulating hormone (α-MSH). The increased α-MSH activates the melanocortin four receptor (MC4R) (140), which in turn mobilizes the sympathetic nervous system, inhibits insulin secretion, enhances hepatic insulin sensitivity (141), and suppresses appetite by modulating NPY/AgRP neurons that promote insulin (142). This may represent a compensatory response to hyperinsulinemia. In transgenic mice with specific overexpression of PYY in pancreatic β-cells, they showed increased β-cell mass and enhanced basal insulin release, further supporting the positive role of PYY in β-cell homeostasis (4, 138).

Exogenous PYY drugs, especially PYY (3-36), showed consistent metabolic improvements in different species and various disease models. In the obesity/diabetes model, long-term infusion of PYY (3-36) through osmotic pumps or long-acting analogues can significantly reduce obesity in gene mutant mice (leptin-deficient mice), db gene mutant mice (leptin receptor-deficient mice), Zucker diabetic fat (ZDF), and Diet-induced obesity (DIO) mice. They improved glucose tolerance and reduced glycated hemoglobin (HbA1c) and fructosamine levels in mice (143, 144). In the high-insulin normal blood glucose clamp experiment, acute infusion of PYY (3-36) can improve the systemic glucose processing rate (M value) of DIO mice, which directly proves its insulin-sensitizing effect (145). In db/db mice, the combined treatment of PYY and GLP-1 significantly enhanced systemic glucose treatment and liver glycogen synthesis with insulin stimulation (107). *In vitro* studies show that PYY (1-36) and PYY (3-36) can promote beta cell proliferation and protect them from STZ-induced apoptosis, and their effect is comparable to or even better than GLP-1. These findings provide key evidence that PYY can improve IR.

### 5.2 Human research evidence

Most cross-sectional studies reported that compared with thin people, the level of PYY on an empty stomach or after meals was reduced, and the PYY secretion response to the standard diet was weakened (41, 146, 147). Similarly, similar PYY secretion defects were observed in T2DM patients and their first-degree relatives (135). These correlations indicate that the lack of PYY may be related to IR and T2DM susceptibility. However, there are inconsistent research results. Some studies have reported that the level of PYY in obese individuals has not changed or even increased (148–151). This variability may stem from methodological factors, group heterogeneity, disease stage, and other considerations. In individuals with normal glucose tolerance, lower fasting PYY levels were associated with higher fasting insulin levels and HOMA-IR (IR index), and negatively associated with HOMA-β (β cell function index). This indicates that low PYY status may be associated with hyperinsulinemia and IR (135, 138). Circulating PYY levels tend to normalize in people who have successfully lost weight, whether through lifestyle intervention or bariatric surgery. It is worth noting that the secretion of PYY and GLP-1 increased significantly after meals after Roux-en-Y gastric bypass surgery (RYGB), which is considered to be one of the key mechanisms for metabolic improvement brought about by the operation (152, 153).

In humans, acute intravenous infusion of PYY(3−36) has consistently been shown to reduce hunger and food intake in a dose−dependent manner (by approximately one−third), an effect observed in both lean and obese individuals (154). This has a positive impact on the long-term control of energy balance. Regarding its acute effect on insulin secretion and sensitivity, the research results are still inconsistent. Some studies have found that it has no significant effect on insulin levels, while others have shown that it may increase insulin sensitivity (118, 144).

In addition, PYY interacts with other treatment modalities. Studies have found that the classic insulin sensitizer metformin can increase circulating PYY levels over the long term (155). This suggests that PYY may partially mediate some of the metabolic benefits of metformin, particularly its mild weight loss and appetite suppression effects. DPP-4 inhibitors treat T2DM by inhibiting the degradation of GLP-1 and GIP. However, DPP-4 is also a key enzyme in the generation of PYY(3-36). Therefore, DPP-4 inhibitors may theoretically reduce the production of PYY(3-36), which could partially offset the benefits brought by GLP-1, such as satiety. This complex interaction warrants further investigation. Although the aforementioned human studies provide important insights into the role of PYY in human metabolism, there are notable inconsistencies within the existing evidence, and these studies themselves have several key limitations that warrant cautious interpretation. For example, conflicting findings regarding whether baseline PYY levels are reduced, unchanged, or elevated in obese individuals may stem from methodological heterogeneity, including differences in the nutritional composition of test meals, blood sampling time points, and the antibody specificity used to detect different PYY isoforms. Additionally, confounding factors such as varying metabolic health statuses of study populations, differences in the duration of obesity, and the presence of comorbidities (e.g., non-alcoholic fatty liver disease) may contribute to divergent secretion patterns. The elevated PYY levels observed in some studies might even represent a compensatory response to severe insulin resistance and hyperphagia rather than an indication of functional sufficiency. More fundamentally, the current body of human evidence shares several common limitations. First, most studies employ cross-sectional designs, which can only establish associations between PYY levels and metabolic phenotypes without proving causality. Second, while acute PYY infusion effectively suppresses appetite, its long-term effects on weight maintenance and insulin sensitivity still lack robust support from large-scale, long-term interventional studies. Third, human research primarily relies on measurements of circulating hormone concentrations, making it difficult to delve into PYY’s receptor signaling mechanisms in specific peripheral tissues, such as the liver, adipose tissue, or skeletal muscle, as animal studies do, thereby limiting our thorough understanding of its mechanistic role in humans. Finally, individual responses to PYY therapy may vary significantly, and the determinants behind this variability (e.g., receptor polymorphisms, blood-brain barrier permeability) remain insufficiently elucidated. Thus, future research requires more rigorously designed prospective studies and long-term clinical trials with detailed phenotypic characterization to clarify PYY’s precise role in human metabolic homeostasis and to define its potential as a therapeutic target and the applicable patient populations.

## 6 The future and prospects of PYY

### 6.1 Potential of PYY as a therapeutic target

The clinical application of PYY(3−36) is limited by its short half−life and dose−dependent side effects such as nausea and vomiting (156). Currently, researchers are developing long-acting, low-side-effect PYY analogs using the following strategies. The half-life of PYY analogs can be extended by PEGylation (polyethylene glycol modification), fatty acid chain modification, or conjugation with albumin (157). For example, PEGylated PYY(3-36) has a half-life extended to 24 hours and does not show toxicity in a rabbit model (157). PYY can also be fused with active fragments of other hormones, such as GLP-1 and glucagon, to develop dual or triple target agonists. For instance, the dual agonist Fc-PYY+Fc-GLP-1 shows synergistic effects on weight loss and improvement of IR in DIO mice and db/db mice, and has stronger beta-cell protective effects (158, 159), while possibly reducing gastrointestinal side effects by lowering the doses of individual components (160, 161). This strategy is grounded in the well-established physiological synergy between PYY and GLP-1. By co-activating Y2 and GLP-1 receptors within a single molecule, these dual agonists aim to recapitulate and potentially enhance the natural, complementary actions of the native hormones on appetite suppression, glycemic control, and β-cell preservation. Oral formulations can also be developed by encapsulating PYY analogs in nanoparticles or using enteric coatings to improve their oral bioavailability. Currently, oral PYY analogs have entered preclinical research stages, with preliminary results showing they can effectively reduce food intake in mice and improve glucose tolerance (156, 162).

The Y2 receptor is the primary target receptor for PYY(3-36). Developing selective Y2 receptor agonists can enhance PYY’s anorexic and insulin-sensitizing effects while reducing actions on other Y receptors (such as Y1 receptor-mediated insulin secretion inhibition). At present, a variety of Y2 receptor agonists have entered phase I clinical trials, and preliminary results show that the food intake of healthy volunteers has decreased significantly (163). Y1 receptors play a crucial role in the protection of pancreatic β-cells. The development of Y1 receptor regulators can improve the function of β-cells, especially in T2DM patients. Studies show that the combined use of Y1 receptor agonists and GLP-1 receptor agonists (GLP-1RA) can induce β-cells into a “rest state” in an obesity-driven diabetes model and promote the recovery of β-cell function (53).

Regulating the proliferation of intestinal L cells and PYY secretion can endogenously increase PYY levels, avoiding the side effects of exogenous administration. Increasing the proportion of fat (especially unsaturated fatty acids) and dietary fiber in the diet can stimulate PYY secretion from L cells. For example, a high-fiber diet can promote L cell proliferation and PYY gene expression by increasing SCFA levels (38, 164). The use of probiotics (such as Bifidobacteria) or prebiotics (such as fructooligosaccharides) can improve the composition of the gut microbiota, increase the abundance of SCFA-producing bacteria, and thereby promote PYY secretion (38, 165). L cell proliferants or PYY secretion enhancers can also be developed, such as TGR5 agonists, which activate TGR5 in intestinal L cells and stimulate the secretion of PYY and GLP-1 (166, 167).

Exogenous PYY is homologous to endogenous pathways and can achieve more natural metabolic regulation without disrupting the gut-brain neural network, theoretically offering better safety. Preclinical and early clinical studies have confirmed that PYY, when used in combination with widely applied GLP-1 receptor agonists, produces a synergistic effect of “1 + 1>2,” significantly reducing total energy intake by about 30% and greatly enhancing satiety (119, 168). Additionally, plasma PYY levels are commonly decreased in obese and T2DM patients, suggesting that PYY supplementation has potential as a causal treatment. Furthermore, in preclinical models of IR-related diseases such as non-alcoholic fatty liver disease (NAFLD/MAFLD) and polycystic ovary syndrome (PCOS), PYY also shows potential in improving hepatic lipid accumulation and metabolic parameters (169).

### 6.2 Challenges faced

The half-life of natural PYY is extremely short (only about 8–12 minutes) (170) and requires frequent drug use, which seriously limits its clinical feasibility. The specific mechanism of PYY (1-36) and PYY (3-36) in IR is still not fully understood, especially in terms of human differences. Future research should focus on selective inhibition or activation of specific subtypes, combined with techniques such as single-cell sequencing and metabolomics, to clarify their tissue-specific effects. PYY regulates metabolism through the intestinal-brain axis and the intestinal-peripheral axis. Nevertheless, the precise neural pathways and signaling molecules involved still need to be further clarified, how PYY regulates the metabolism of the liver and skeletal muscles through the central nervous system, and how the intestinal microbiota participates in this process. At the same time, exogenous PYY administration often causes gastrointestinal side effects such as nausea and vomiting, limiting its dose and long-term use. Long-term drug desensitization may cause Y2 receptor down-regulation or signal desensitization, thus weakening its long-term efficacy. Factors such as obesity, diabetes status, and genetic background can also affect the plasma level and receptor sensitivity of PYY, resulting in significant differences in the efficacy of different patients. The current methods of subcutaneous injection or continuous infusion are not conducive to long-term management, and the development of a drug administration system with high patient compliance (such as oral or weekly injection) is still a major challenge.

### 6.3 Future research directions

In order to meet these challenges, the future development path will focus on molecular design, delivery innovation, and precision medicine. Polyethylene glycolization, Fc fusion, fatty acid chain modification, albumin binding, and other mature technologies can significantly extend the cycle time, laying the foundation for convenient drug use. The design of analogues with higher selectivity and effectiveness against Y2 receptors can maximize the therapeutic effect and minimize side effects. In addition, the development of multi−target agonists that simultaneously activate PYY receptors (Y2R) and other metabolism−related receptors (e.g., GLP−1 receptors) has become a focus of current drug discovery. GLP-1/PYY double agonists and GLP-1/GIP/Y2 triple agonists are designed to replicate synergistic effects within a single molecule, indicating that they have stronger comprehensive metabolic regulation potential. The industry regards these as the key directions of the “next wave” of metabolic drugs. Clinical transformation is steadily advancing, and pioneering compounds such as CIN-110 (long-acting PYY3–36 analogue) have successfully entered clinical trials. The phase I study proved good safety, prolonged pharmacokinetics, and significant weight loss trends (171). In addition, more candidate drugs such as NNC0165-1875 (a GLP-1/PYY double agonist) have entered clinical evaluation, providing empirical evidence for their efficacy.

Innovative drug administration and precision medical treatment, and explore more convenient ways, such as long-acting subcutaneous injection, oral or nasal administration, in which nasal administration shows minor gastrointestinal side effects. Patients were stratified using biomarkers such as plasma PYY levels, Y2 receptor expression, and SCFA to identify patient subgroups most likely to benefit and enable precision therapy. Scientific control of side effect strategies, research confirms that the rapid peak of drug concentration is a key factor leading to gastrointestinal adverse reactions. The incidence of these adverse events can be significantly reduced by adopting a slow-release molecular design (such as polyethylene glycolization) and a gradual dose increment scheme.

## 7 Conclusion

PYY, a hormone derived from the intestine, has developed from being originally considered a simple “anorexic peptide” to a core participant in our understanding of the intestinal-brain-peripheral metabolic axis regulation network. Its role in improving insulin resistance is multifaceted and multi-targeted. It provides indirect benefits by mediating central appetite suppression and weight loss, and finely regulates insulin secretion and sensitivity by directly acting on the islet, liver, and adipose tissue. A large number of preclinical and clinical studies support the view that PYY system dysfunction is an important part of the pathogenesis of obesity, insulin resistance, and type 2 diabetes. Restoring or enhancing PYY signal conduction can effectively reverse these metabolic abnormalities. Although the direct conversion of PYY to drugs faces challenges such as short half-life and gastrointestinal side effects, these challenges are being gradually overcome through the development of long-acting analogues, multi-target agonists, and reasonable combined treatment strategies. In the future, with the development of long-acting PYY analogs, selective Y receptor modulators, and gut L-cell regulatory strategies, combined with in-depth mechanistic studies and large-scale clinical trials, PYY is expected to become a novel therapeutic target for insulin resistance and related metabolic disorders.
